# Validation of the Gaslini Pediatric Nursing Score© to Assess Pediatric Patients’ Nursing Complexity

**DOI:** 10.3390/children13101370

**Published:** 2026-10-09

**Authors:** Roberta Da Rin Della Mora, Simona Calza, Roberta Tirone, Ilaria Artuso, Stefano Parodi, Silvia Rossi, Giulia Ottonello, Giuseppe Minniti Caracciolo, Nicoletta Dasso, Silvia Scelsi

**Affiliations:** 1 Center for Research in Nursing and Health Professions, Health Professions Directorate, IRCCS Istituto Giannina Gaslini, 16147 Genova, Italy; robertadarindellamora@gaslini.org; 2 Health Profession Directorate, IRCCS Istituto Giannina Gaslini, 16147 Genova, Italy; simonacalza@gaslini.org (S.C.); robertatirone@gaslini.org (R.T.); silviarossi@gaslini.org (S.R.); giuliaottonello@gaslini.org (G.O.); giuseppeminniti@gaslini.org (G.M.C.); nicolettadasso@gaslini.org (N.D.); silviascelsi@gaslini.org (S.S.); 3 Epidemiology and Biostatistics Unit, Scientific Directorate, IRCCS Istituto Giannina Gaslini, 16147 Genova, Italy; stefanoparodi@gaslini.org

**Keywords:** hospital administration, hospitalized child, nursing assessment, nursing complexity, Nursing Patient Classification Systems, pediatric hospital, pediatric nursing, validation study

## Abstract

**Highlights:**

**What are the main findings?**

- Statistical validation of the Gaslini Pediatric Nursing Score© (GPNS©), used to assess pediatric patients’ nursing complexity in low- and medium-intensity care units of a tertiary pediatric teaching hospital, demonstrated substantial inter-rater reliability, short completion time, and very high perceived ease of use. Exploratory Factor Analysis (EFA) provided evidence consistent with a unidimensional factor structure, supporting the tool’s construct validity.
- The fair agreement with clinical judgment does not allow considering concurrent validity as established, and the paucity of patients classified within the high-complexity level was not sufficient to consider validation for this subgroup as established.

**What are the implications of the main findings?**

- The GPNS© constitutes a reliable, non-time-consuming, and easy-to-use tool for assessing nursing complexity among hospitalized pediatric patients in low- and medium-intensity care units within its development setting.
- Further studies comparing the GPNS© with an externally validated criterion remain necessary. In addition, further research is also needed to assess a larger, representative sample of patients classified at the high-complexity level.

**Abstract:**

**Background/Objectives:** Aligning care delivery with patient needs is essential for the quality of care. As nursing needs vary in complexity, Nursing Patient Classification Systems (NPCSs) are critical to guide patient allocation to appropriate care settings. The “Gaslini Pediatric Nursing Score©” (GPNS©) was developed to assess nursing complexity in pediatric inpatients. Following face and content validity testing, this study aimed to validate the GPNS© by assessing its inter-rater reliability, concurrent validity using clinical judgment as the reference standard, construct validity, completion time, and perceived ease of use. **Methods**: A prospective observational validation study was conducted at a tertiary pediatric teaching hospital. All pediatric patients (from birth to <18 years) admitted to the 17 low- and medium-intensity care units during the study period were included. Data were collected via digital forms by shift nurse pairs. **Results**: Overall, 389 nurses participated; 192 patients were assessed in pairs, yielding 814 valid forms. Inter-rater reliability was substantial (Cohen’s kappa = 0.785), whereas concurrent validity showed fair agreement (Cohen’s kappa = 0.300), with disagreements occurring predominantly between adjacent complexity levels (weighted Cohen’s kappa = 0.523). Exploratory Factor Analysis (EFA) isolated a single latent variable, conceptualized as “nursing complexity”. The mean completion time was 1.8 min, with a 96.7% positive rating for perceived ease of use. **Conclusions**: Within its development setting, the GPNS© demonstrated substantial inter-rater reliability, short completion time, and very high ease of use, with EFA providing evidence consistent with construct validity. However, both the fair concurrent validity and the paucity of patients classified within the high-complexity level were not sufficient to consider validation of the GPNS© as fully established. Further studies against an external criterion and on larger samples of high-complexity patients remain necessary.

## 1. Introduction

Providing the appropriate level of care to each patient is essential to ensure healthcare quality [1]. A quality health service is defined as one “which organizes resources in the most effective way to meet the health needs of those most in need, for prevention and care, safely, without waste and within higher level requirements” [2]. Some authors have proposed a conceptual framework to understand the relationship between need, demand, and supply in healthcare. In particular, they linked healthcare needs to appropriate care, defined either as cost-effective care or, when cost-effectiveness is unknown, as “the right care provided in the right place and at the right time” [3].

In nursing, the concept of “need” remains central to both clinical practice and research and is directly tied to the latest internationally recognized definition of nursing provided by the International Council of Nurses [4]. Because patients’ needs vary in type and complexity, the required nursing care differs accordingly. Therefore, patient assessment is critical to provide the information needed to drive patient allocation, assignment, care, and discharge planning [5,6].

In this context, patient classification—defined as “a categorization of patients according to some assessment of their nursing care requirements over a specified period of time” [7]—implemented through a Patient Classification System [5], is recognized as a key component of patient care management [5,8,9]. To classify patients according to various aspects of nursing care, several Nursing Patient Classification Systems (NPCSs) have been developed, including some specifically designed for pediatric settings [10]. Over the years, the adoption of NPCSs has become increasingly relevant as hospitals have undergone major organizational transformations [9].

When our tertiary pediatric teaching hospital began planning the transition from a traditional unit-based model to an intensity-of-care model, the need for an NPCS to support patient allocation based on nursing complexity became apparent. The Health Professions Directorate therefore commissioned a research team of nurse scientists to identify an existing NPCS—or to develop a new one if no suitable tool was available—to assess pediatric patients’ nursing complexity (henceforth “nursing complexity” or “complexity”) and guide their allocation to low- and medium-intensity care units. For this purpose, the research team adopted the definition of nursing complexity proposed by Richards and Borglin [11] (p. 531): “Nursing’s complexity is such that it can be seen as the quintessential ‘complex intervention’—defined as an activity that contains a number of component parts with the potential for interactions between them which, when applied to the intended target population, produces a range of possible and variable outcomes.” Based on this *a priori* definition, a narrative review was conducted to provide an overview of validated NPCSs assessing nursing complexity, as defined, in hospitalized pediatric patients. However, this review yielded no results [10].

To address this gap, the research team designed a project to develop an NPCS based on: (a) its intended purpose (guiding the allocation of hospitalized pediatric patients to the appropriate nursing care setting); (b) its core focus (assessing the nursing complexity of hospitalized pediatric patients—i.e., the complexity of both their nursing needs and the required nursing care); (c) its conceptual framework (Henderson’s Need Theory) [12]; (d) the adopted definition of “nursing complexity” [11] (p. 531); and (e) an appropriate scientific methodology [13].

The research team comprised five senior nurses, each with over 20 years of clinical experience; four of them were pediatric nurses, and two held a PhD in Nursing. A team of epidemiologists and statisticians also collaborated on the project.

As recommended in the literature, the NPCS was designed to include only the elements essential for a valid classification of hospitalized pediatric patients, thereby remaining time-efficient and easy to use [13,14]. The resulting NPCS relies on an assessment tool named “Gaslini Pediatric Nursing Score©” (GPNS©) [15].

However, tool development represents only the first step toward clinical implementation. Any newly developed tool must undergo rigorous statistical validation before use [14,16] to establish its reliability and validity, thereby ensuring its dependable application in practice [7,16,17]. Inter-rater reliability is typically assessed by evaluating the degree of agreement between scores assigned independently by different raters to the same subject [17]. Accordingly, in this study, inter-rater reliability was quantified as the degree of agreement between the GPNS© complexity levels assigned independently by two different nurses to the same patient. Conversely, concurrent validity is typically assessed by comparing the new tool’s results with those of an established, validated tool measuring the same latent variable [17]. In the absence of a comparable tool [18], the only feasible option was to consider expert clinical judgment—although subject to potential bias [19]—as a pragmatic reference standard [20]. For this study, clinical judgment was defined as “an interpretation or conclusion about a patient’s needs, concerns, or health problems, and/or the decision to take action (or not), use or modify standard approaches, or improvise new ones as deemed appropriate by the patient’s response” [21] (p. 204). Thus, in the absence of an externally validated tool to serve as a gold standard at the time of the study, we decided to quantify concurrent validity as the degree of agreement between a nurse’s overall clinical judgment complexity level and the GPNS© complexity level assigned to the same patient by the same nurse. Additionally, inter-rater agreement between the clinical judgment levels provided independently by nurse pairs was assessed.

Finally, mean completion time and perceived ease of use provide valuable insights into the tool’s time efficiency and user-friendliness [14].

Therefore, this study aimed to validate the GPNS© by assessing its inter-rater reliability, concurrent validity assuming clinical judgment as a pragmatic reference standard, construct validity, mean completion time, and perceived ease of use when administered by nurses or pediatric nurses (henceforth “nurses”) in low- and medium-intensity care units.

## 2. Materials and Methods

### 2.1. Study Design

We conducted a prospective observational validation study. The study was reported in accordance with the Strengthening the Reporting of Observational Studies in Epidemiology (STROBE) guidelines. The completed STROBE checklist is provided as Supplementary Material (Table S1).

### 2.2. Setting

The study was conducted across all 17 low- and medium-intensity care units at a multispecialty tertiary pediatric teaching hospital in Northern Italy.

### 2.3. Population

According to DeVellis [17], at least five subjects per item are required to assess the dimensionality of a scale; thus, at least 200 subjects were needed for the present study. Based on the expected number of admitted patients in our setting, a consecutive cohort of pediatric patients (from birth to <18 years) admitted to the 17 low- and medium-intensity care units during the one-month study period was included. Patients could be assessed on multiple occasions, provided that the assessments occurred on different days.

### 2.4. Data Collection Tool

The GPNS© was developed to assess the nursing complexity of hospitalized pediatric patients based on the complexity of their nursing needs and the care required, in order to allocate them to the appropriate nursing care setting.

#### 2.4.1. GPNS© Development Process

The research team developed the tool through a multi-step process.

First, ten “core” nursing needs were identified based on Virginia Henderson’s Need Theory [12] and an extensive literature review. For each identified need (domain), a provisional set of items representing nursing interventions was established. Four levels of nursing complexity were defined: low, medium-low, medium-high, and high, corresponding to white, green, yellow, and red color codes, respectively. These color codes were used solely as labels, without any operational implications.

Two sequential Delphi studies were then conducted to assign each item—representing a nursing intervention or group of interventions—to one of the four complexity levels [22]. A panel of 40 expert pediatric nurses providing direct patient care across 20 units was recruited. Panelists received information about the project and the adopted definition of nursing complexity before assigning each item to a complexity level. In addition, they were asked to provide comments on the items, if any. Qualitative feedback from the first Delphi study prompted modifications to the provisional item set. For example, similar items within the same complexity level were merged (e.g., “total parenteral nutrition” and “partial parenteral nutrition” were combined into “total and partial parenteral nutrition”), and “no intervention” items were added to represent the lowest complexity level. Because these revisions substantially altered the tool, a second Delphi study was conducted. This study involved a new panel of 40 expert pediatric nurses from the same 20 units, 38 of whom participated.

This process yielded a preliminary version of the GPNS©, ready for face and content validity testing [23].

#### 2.4.2. GPNS© Face and Content Validity Assessment

The face and content validity of both the individual items and the overall tool were then assessed by a panel of six experienced nurses who were involved in neither the research team nor the Delphi studies [23]. Minor adjustments were made based on their recommendations, resulting in a final 41-item tool. Content validity results were as follows: mean rater proportion of 0.99; Item-Content Validity Index (I-CVI) of 0.99 (range: 0.83–1.00); Scale-Content Validity Index, Universal Agreement (S-CVI/UA) of 0.93; and Scale-Content Validity Index, Average (S-CVI/Ave) of 0.99. These results indicated strong content validity [23,24] prior to psychometric testing.

#### 2.4.3. GPNS© Final Version

The final 41-item version of the GPNS© was previously published elsewhere [15]. By design, the patient’s overall nursing complexity level on the GPNS© is determined by the highest complexity level assigned across any of the 10 core needs: (1) Breathing; (2) Cardiocirculatory function; (3) Consciousness and behavior; (4) Pain management; (5) Monitoring; (6) Education, support, and communication; (7) Feeding and hydration; (8) Urinary excretion; (9) Intestinal excretion; and (10) Mobilization [15].

For the present study, the final version was digitized via the LimeSurvey© (Hamburg, Germany) platform. The content of the digital version was identical to the published version. However, while the paper format provided a compact layout offering a global overview of all domains and items, the digital interface presented the domains in a sequential, step-by-step manner. To ensure data completeness, the application included mandatory fields for each domain prior to submission, but it did not feature any interface prompts or automated suggestions that could have influenced the nurses’ complexity level assignment.

### 2.5. Study Procedure

Once the 17 low- and medium-intensity care units were identified, their respective head nurses were contacted to present the study protocol and request formal participation. Concurrently, all hospital healthcare professionals were invited to an institutional presentation of the project.

Training sessions were then conducted by the research team for the nurses of the participating units. These sessions covered the project background, the tool’s development process, and the practical application of the GPNS© through guided discussions on both real and simulated clinical scenarios. Interactive small-group workshops were also organized, allowing participants to practice applying the tool to clinical cases from their own daily practice. Periodic reminders were sent to nurses who had not yet attended the training and workshops, aiming to achieve an overall participation rate of at least 90% across the involved units.

To minimize novelty bias and foster familiarity, nurses were given access to the digital form for one month prior to data collection. Additionally, eight nurses who completed all training sessions were recruited on a voluntary basis as clinical champions. Acting as a peer-to-peer support network, these nurses assisted colleagues during the one-month pre-implementation phase as they familiarized themselves with the digital tool. This phase enabled nurses to gain proficiency with the digital platform, clarify operational doubts, and resolve application issues alongside the clinical champions.

The digital tool was accessible via computer in each unit using personal login credentials through a dedicated link on the hospital intranet homepage.

Since patients could be assessed on multiple occasions, nurses were blinded to the patients’ overall GPNS© complexity levels to prevent this information from influencing subsequent assessments.

The study findings were ultimately disseminated to all involved nurses.

### 2.6. Data Collection

Data collection was conducted digitally by pairs of shift nurses, each possessing a minimum of six months of clinical experience in their respective unit. These nurses were selected due to their direct involvement in patient care.

To assess inter-rater reliability, each nurse pair independently assessed the nursing complexity of the same patient using the GPNS© once per shift, following the nursing handover. These assessments were conducted either simultaneously or within a maximum interval of 2 h.

To assess concurrent validity, prior to completing the GPNS©, both nurses independently assigned an overall nursing complexity level to the patient based on their clinical judgment. This global assessment used a 4-point Likert scale ranging from 1 (lowest complexity) to 4 (highest complexity).

Upon completing each digital assessment, nurses recorded the time required to complete the tool and rated its perceived ease of use on a 4-point Likert scale (1 = very easy, 2 = fairly easy, 3 = fairly difficult, 4 = very difficult). Patients’ dates of birth were automatically collected during each submission.

Data collection occurred simultaneously across all 17 low- and medium-intensity care units during August 2020. Upon completion, data were exported from the LimeSurvey© platform into Microsoft Excel^®^ 2007 for analysis.

### 2.7. Data Analysis

Statistical analyses were conducted using Stata software, version 18.0 MP (StataCorp, College Station, TX, USA).

Continuous variables were described using the mean, median, range, and standard deviation (SD), while categorical variables were summarized using absolute and relative frequencies. These descriptive statistics were used to describe the characteristics of both the assessed pediatric patients and the participating nurses, as well as the completion time and perceived ease of use of the GPNS© tool.

Inter-rater reliability and concurrent validity were assessed using Cohen’s kappa index [25]. Both unweighted and weighted kappa (using quadratic weights) were computed and their corresponding 95% confidence intervals (95% CI) were estimated, adjusting for repeated measures using 2000 clustered bootstrap samples.

Heterogeneity in agreement across different units was assessed using Cochran’s *Q* test. A proportion of agreement of 75% (kappa > 0.75) was predefined as the threshold for optimal concordance [25].

As the GPNS© is a new tool, its dimensionality was assessed by Exploratory Factor Analysis (EFA), including all domains. Following the assignment of the complexity level resulting from the item assessment, each domain was analyzed as an ordinal scale, assigning a value of 1 to the low complexity level (white), 2 to the medium-low (green), 3 to the medium-high (yellow), and 4 to the high level (red). All the valid digital forms, completed by the two nurses of each pair, were included. Data suitability for EFA was evaluated using the Kaiser–Meyer–Olkin (KMO) measure. Factors were extracted via the principal factors method followed by orthogonal varimax rotation. A visual inspection of the corresponding eigenvalues was performed using a scree plot. Values greater than 1.0 were considered indicative of the presence of latent variables, according to the Kaiser criterion [17].

### 2.8. Risk of Bias

To minimize information bias, nurses received standardized training on the GPNS© prior to data collection, followed by a one-month familiarization period supported by a peer-to-peer network of clinical champions. During this phase, user queries were systematically addressed, clarified, and compiled into an operational manual featuring frequently asked questions [14].

To reduce observer bias, nurse pairs assessed the same patient independently and without prior discussion. These paired assessments were conducted within a 2-h window to minimize the likelihood of relevant changes in the patient’s nursing needs.

Additionally, nurses were asked to record their overall clinical judgment of nursing complexity prior to completing the GPNS© to prevent the tool’s complexity level from influencing their clinical judgment.

To evaluate potential same-rater bias, the difference between the observed Cohen’s kappa index and that obtained through the bootstrap procedure was calculated.

### 2.9. Ethical Considerations

The study protocol, in the form described in the present paper, was approved by the hospital’s Institutional Review Board (protocol code 117/2017).

Participating nurses were informed about the study objectives via an introductory statement displayed upon accessing the LimeSurvey© platform prior to data collection. The completion of the GPNS© digital forms was considered implicit informed consent.

Informed consent from pediatric patients and their legal guardians was waived by the Institutional Review Board, as completion of the GPNS© was based on data already routinely collected during standard nursing care without altering clinical practice or patient management, and GPNS© results were not used to alter the nursing care provided. For this reason, no formal opt-out mechanism for parents or legal guardians—to object to the use of the data for research purposes—was established.

To ensure maximum security, data integrity, and regulatory compliance, the GPNS© was hosted on a dedicated LimeSurvey© server deployed within the enterprise infrastructure farm of Liguria Digitale (the in-house IT provider for the Liguria Regional Government). The application ran on a high-availability (HA) VMware virtual machine architecture and was shielded by perimeter security systems managed by Liguria Digitale, which enforced geo-blocking to restrict server access. All user interactions were encrypted and routed via an NGINX reverse proxy using the HTTPS protocol, ensuring that direct exposure of the application server to the public network was entirely avoided. From an administrative and operational standpoint, server-level access was restricted exclusively to IT personnel from the hospital. Data integrity and availability were maintained through automated nightly backups, alongside monthly log rotations. Nurses accessed LimeSurvey© using randomly generated, 15-character unique identifier codes provided by the same IT personnel. Finally, upon the expiration of the declared 15-year retention period, data deletion will be performed by dropping the database.

Data were strictly pseudonymized, and findings were analyzed exclusively in aggregate form. No direct patient identifiers were recorded, though date of birth was collected to calculate patient age and to pair the independent nurse assessments for inter-rater reliability analysis. No re-identification key was created. Although nurses accessed the LimeSurvey© platform using individual alphanumeric credentials, the research team had no access to the key linking credentials to individual identities, ensuring participant confidentiality.

All electronic data exported from the LimeSurvey© platform were stored on a password-protected, encrypted hard drive accessible to and managed solely by the data manager. System authentication and access controls prevented unauthorized access, restricting data handling to the data manager, who was also the study statistician.

All procedures adhered to the European Union General Data Protection Regulation (GDPR, EU 2016/679). Data will be retained securely for 15 years following study completion, after which all digital records will be permanently deleted.

## 3. Results

### 3.1. Participant Characteristics

A total of 389 nurses participated in the study. Of these, 364 (93.6%) completed the training sessions and small-group interactive workshops, while the remaining 25 (6.4%) were supported through peer-to-peer clinical champion guidance during the one-month pre-implementation phase. The vast majority of participating nurses (*n* = 386, 99.2%) were female, with a mean age of 40.1 years (SD = 8.5; median = 39; range = 24–57); 375 (96.4%) were pediatric nurses and 9 (2.3%) were general nurses (1.3% missing data).

During the study, 192 pediatric patients were assessed, with a mean age of 5.4 years (SD = 5.1; median = 5; range = 0–16).

### 3.2. Descriptive Analyses

A total of 1076 digital forms were completed. Among these, 230 (21.4%) could not be paired and were subsequently excluded from the analysis, leaving 846 forms. The reasons for non-pairing were missing information on the date and time of form completion due to a malfunction of the electronic registration system in two cases, and the lack of a form completed within the timeframe defined for data collection (two hours) for the remaining forms.

**Figure 1:**
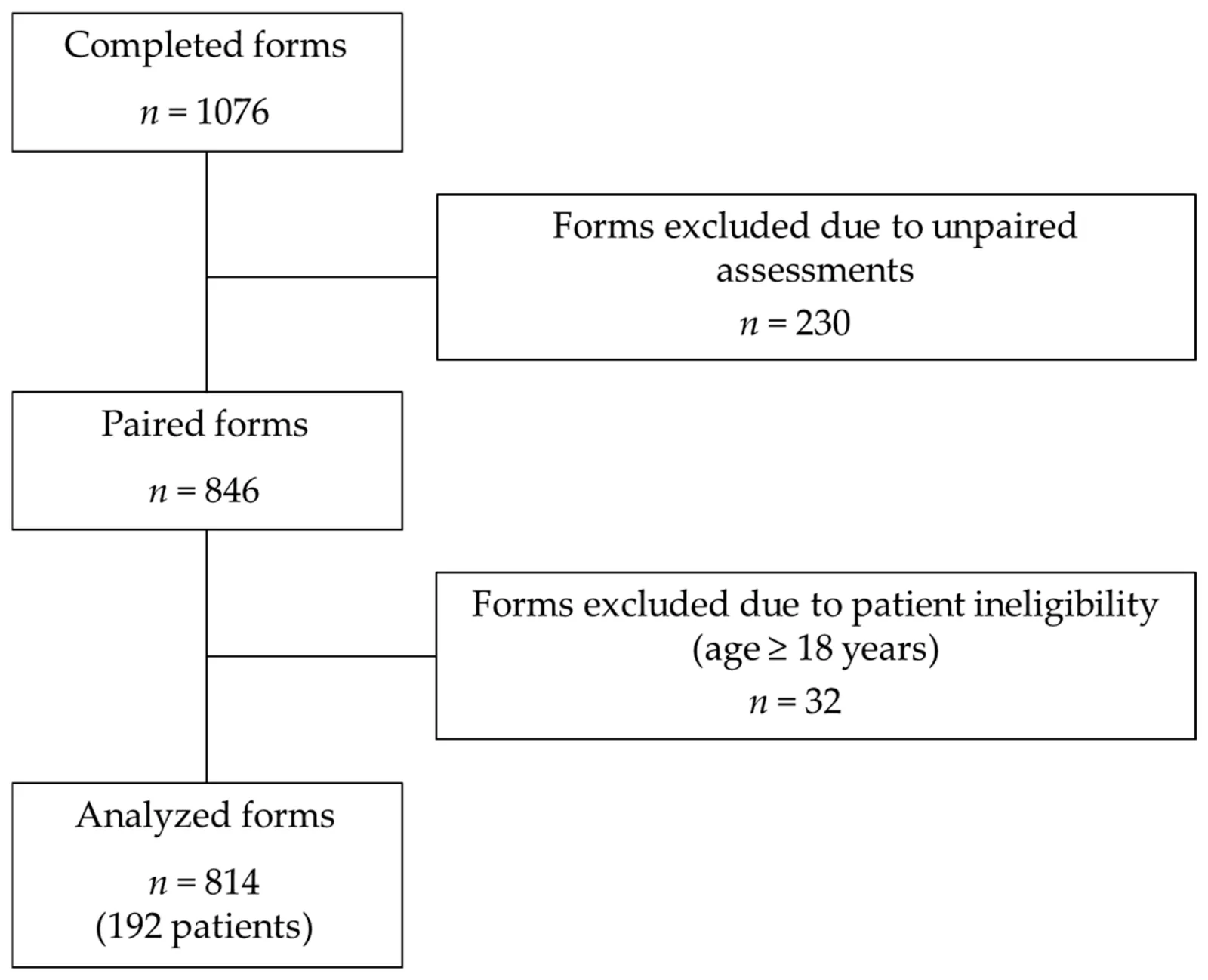
Flow diagram of the digital form selection and inclusion process for analysis.

Thirty-two forms were related to non-eligible patients (aged 18 years or older) and were also excluded. Accordingly, 814 forms were available for analysis, representing 407 paired independent assessments performed on 192 unique pediatric patients (Figure 1).

**Figure 2:**
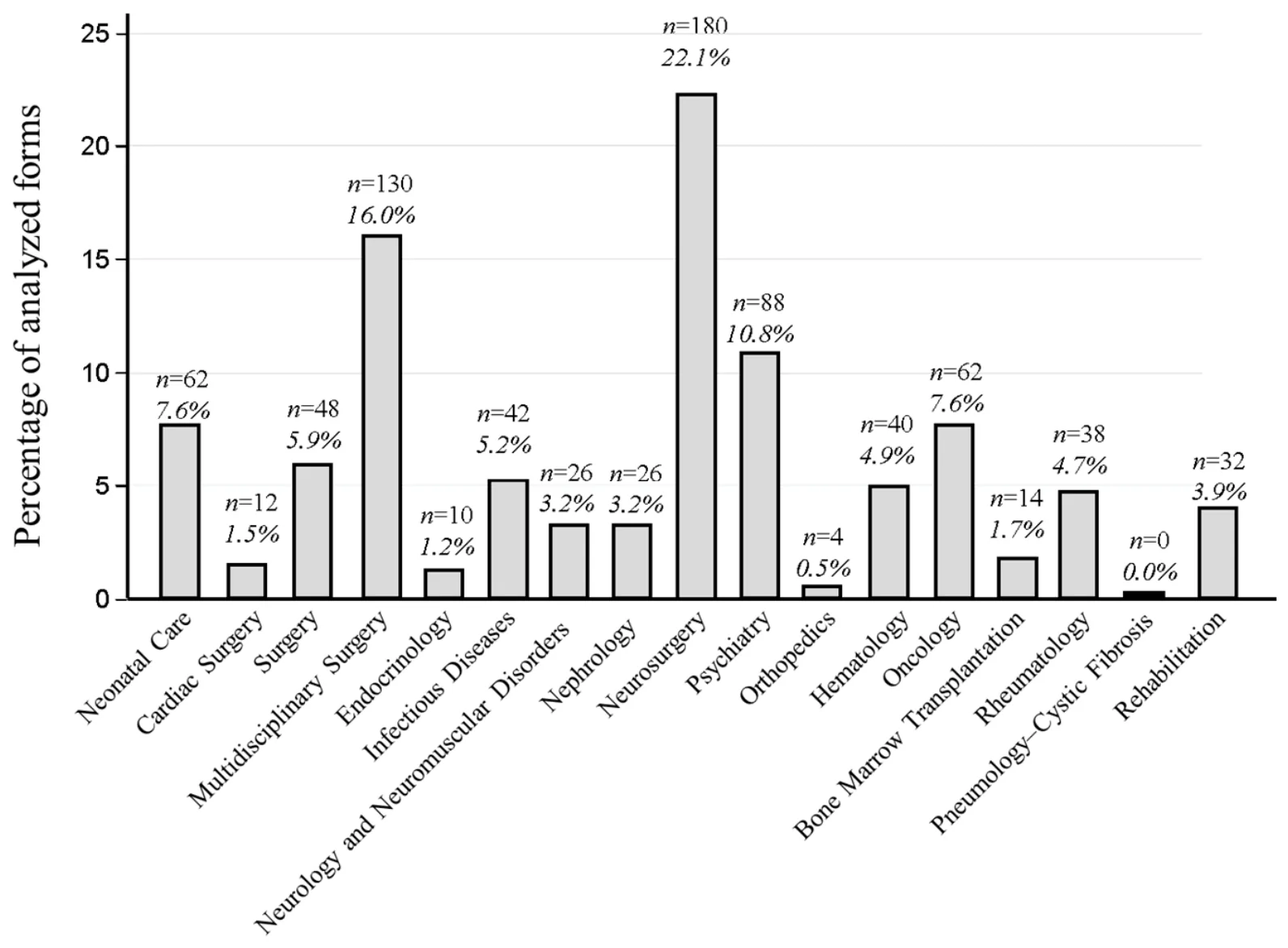
Distribution of analyzed forms across participating units (*n* = 814).

Although 17 units participated, one unit (Pneumology–Cystic Fibrosis) yielded no paired forms; thus, the final analysis was based on 16 units. The number of valid paired forms to analyze varied widely across units, ranging from 4 in Orthopedics to 180 in Neurosurgery (Figure 2).

The analysis of the 230 unpaired forms highlighted a different distribution by unit, with more than 60% of forms originating from the Neonatal Care (*n* = 52, 22.6%), Cardiac Surgery (*n* = 49, 21.3%), and Neurology and Neuromuscular Disorders (*n* = 44, 19.1%) units.

The number of assessments per nurse ranged from 1 to 26 (mean = 8.1, SD = 5.2), while the number of assessments per patient ranged from 1 to 7 (mean = 2.1, SD = 1.5). The vast majority of patients remained in the same unit during the study period, except for 10 subjects who were evaluated in two different units during their hospitalization. Specifically, three patients were evaluated in both Hematology and Oncology; one in Hematology and Bone Marrow Transplantation; one in Oncology and Infectious Diseases; one in Neurosurgery and Infectious Diseases; one in Multidisciplinary Surgery and Infectious Diseases; one in Surgery and Multidisciplinary Surgery; one in Neurosurgery and Psychiatry; and one in Surgery and Oncology.

The white level (low complexity) was reported in 28.5% (*n* = 232) of cases, the green level (medium-low complexity) in 44.5% (*n* = 362), the yellow level (medium-high complexity) in 26.4% (*n* = 215), and the red level (high complexity) in 0.6% (*n* = 5). The distribution among unpaired forms was quite similar, except for a higher prevalence of high complexity levels (red, 3.9%).

### 3.3. Inter-Rater Reliability

The agreement between the results of the GPNS© assessment by the nurse pairs is reported in Table 1. The unweighted Cohen’s kappa index was 0.785 (95% CI: 0.700–0.870, adjusting for repeated measures by operator), while weighted kappa index was 0.839 (95% CI: 0.775–0.904). The corresponding biases (difference between observed kappa indices and their corresponding bootstrap estimates) were negligible in both cases (−0.34% and −0.27%, respectively). Analysis adjusted for repeated measures on the same patient provided similar results: 95% CI: 0.729–0.841 for unweighted kappa index, and 0.788–0.891 for weighted kappa index. Because data were too sparse to allow for a clustered bootstrap by patient and operator, a sensitivity analysis was performed by restricting the dataset to either the first occurrence per operator, adjusting for patient, or the first occurrence per patient, adjusting for operator. In detail, unweighted kappa indices were 0.806 (95% CI: 0.697–0.915, bias = −0.18%) and 0.693 (95% CI: 0.566–0.812, bias = −0.51%), respectively. Weighted kappa indices were 0.893 (95% CI: 0.829–0.957, bias = −0.20%) and 0.778 (95% CI: 0.678–0.877, bias = −0.46%), respectively.

Disagreements occurred predominantly between adjacent complexity levels (e.g., white vs. green, green vs. yellow; Table 1).

At the individual domain level, agreement evaluated by unweighted Cohen’s kappa index exceeded the predefined 0.75 threshold in five domains: Feeding and hydration (0.824), Pain management (0.805), Monitoring (0.808), Urinary excretion (0.788), and Mobilization (0.768). Five domains yielded a kappa index below 0.75: Breathing (0.693), Intestinal excretion (0.732), Consciousness and behavior (0.648), Education, support and communication (0.635), and Cardiocirculatory function (0.582). A similar pattern was observed when analyzing the corresponding weighted kappa index, with the exception of Urinary excretion, which showed a decrease (weighted kappa index 0.669). The other figures were: Feeding and hydration 0.873, Pain management 0.779, Monitoring 0.832, Mobilization 0.817, Breathing 0.711, Intestinal excretion 0.670, Consciousness and behavior 0.616, Education, support and communication 0.660, and Cardiocirculatory function 0.524.

**Figure 3:**
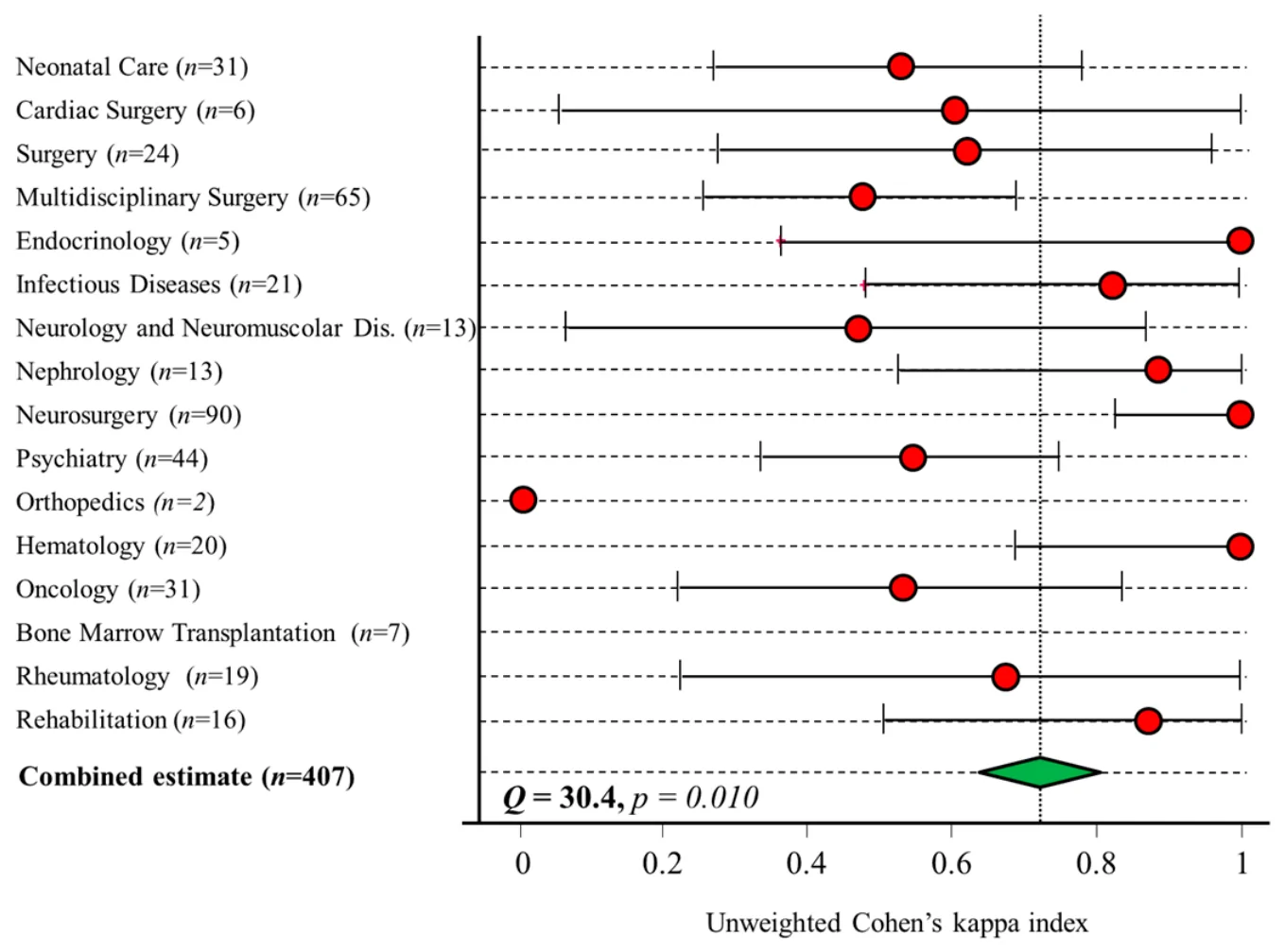
Forest plot of inter-rater agreement (unweighted Cohen’s kappa) across clinical units. Circles and horizontal lines represent the individual unit estimates and their 95% confidence intervals, respectively. The diamond at the bottom indicates the overall pooled estimate and its 95% confidence interval. *Q* = Cochran’s *Q* test statistic for heterogeneity. The Cohen’s kappa index was not evaluable for the Bone Marrow Transplantation unit due to zero variance (identical ratings across all paired assessments).

Significant heterogeneity in inter-rater agreement was observed across clinical units, ranging from 0 in the Orthopedics unit to 1 in the Endocrinology and Hematology units (Cochran’s *Q* = 30.4, *p* = 0.010; Figure 3). When the analysis was repeated using the weighted Cohen’s kappa index, heterogeneity was reduced and no longer statistically significant (*Q* = 16.6, *p* = 0.361).

Inter-rater agreement for overall clinical judgment alone between the two nurses in each pair, measured by the unweighted Cohen’s kappa index, was 0.706 (95% CI: 0.601–0.812, adjusting for repeated measures within operators). The corresponding weighted kappa index was 0.776 (95% CI: 0.676–0.877; Table 2).

Adjusting for repeated measures within patients, the corresponding 95% CIs were 0.637–0.775 and 0.708–0.845. The corresponding biases (difference between observed and estimated kappa indices via bootstrap) were negligible in all models (absolute value < 1.0%).

Restricting the analyses to the first occurrence per operator, kappa indices were almost unchanged (unweighted kappa index: 0.707, 95% CI: 0.584–0.830; weighted kappa index: 0.778, 95% CI: 0.668–0.887). When the first occurrence per patient was considered, the corresponding figures were: 0.653, 95% CI: 0.512–0.793, and 0.784, 95% CI: 0.693–0.877, respectively.

### 3.4. Concurrent Validity

Agreement between overall nursing clinical judgment and the GPNS© results, adjusting for repeated measures within operators, was 0.300 (95% CI: 0.223–0.376) for the unweighted Cohen’s kappa index and 0.523 (95% CI: 0.454–0.593) for the weighted kappa index (Table 3).

When the analysis was performed adjusting for patient-level clustering, similar results were observed (95% CI: 0.231–0.369 for the unweighted kappa index and 95% CI: 0.448–0.598 for the weighted kappa index). Consistent results were also obtained after restriction to the first occurrence either per operator (unweighted kappa index: 0.273, 95% CI: 0.129–0.418; weighted kappa index: 0.502, 95% CI: 0.368–0.635) or per patient (unweighted kappa index: 0.261, 95% CI: 0.139–0.383, bias = −1.9%; weighted kappa index: 0.519, 95% CI: 0.416–0.621, bias = −1.0%). Model biases were negligible in all analyses (<2%).

Discrepancies were primarily driven by four domains: Monitoring, Pain management, Mobilization, and Education, support, and communication.

On average, nursing complexity levels classified by the GPNS© were higher than those assigned via subjective clinical judgment. In this case as well, disagreements occurred predominantly between adjacent complexity levels (e.g., white vs. green, green vs. yellow).

### 3.5. Dimensionality

**Figure 4:**
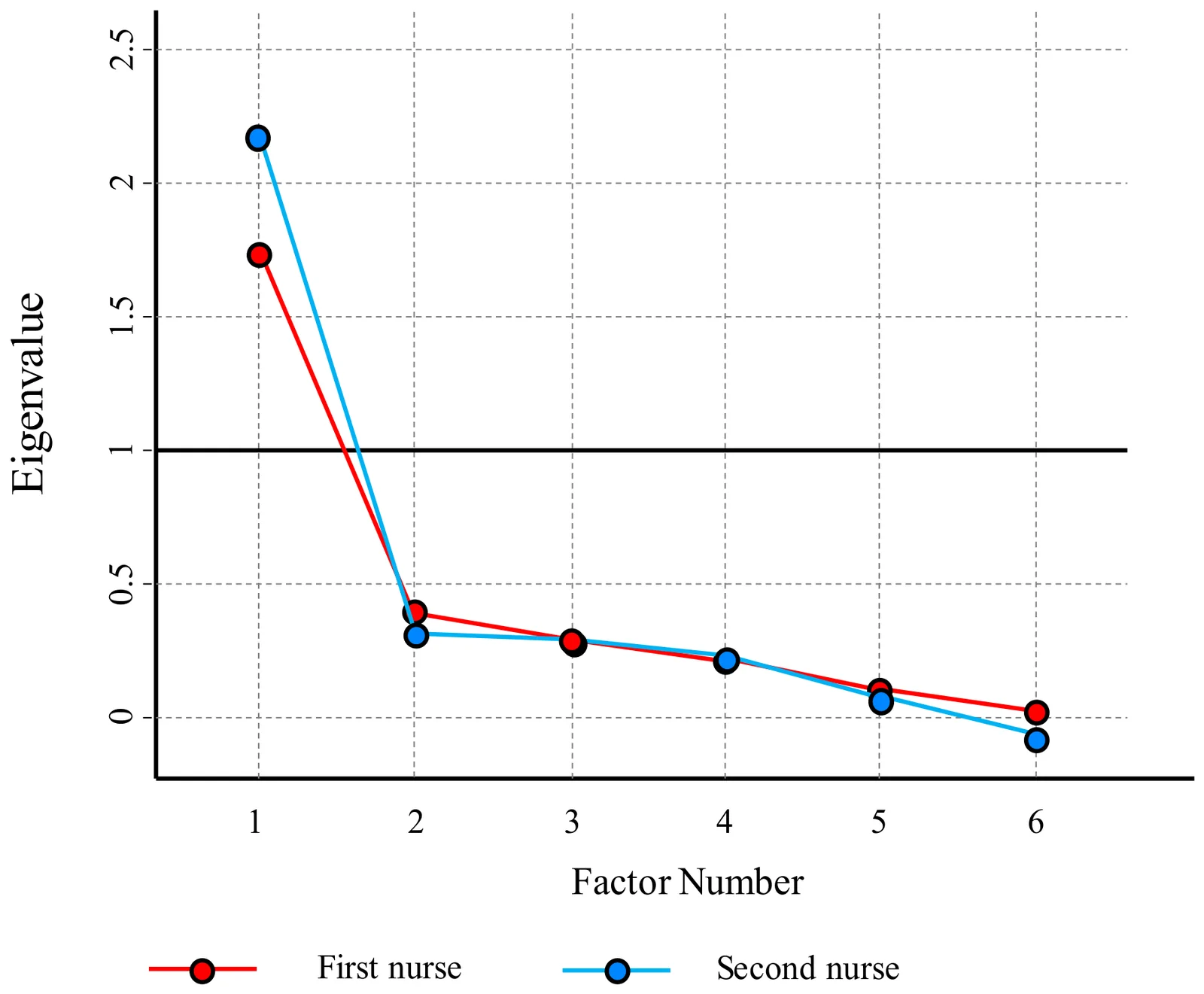
Scree plot of the Exploratory Factor Analysis for the first and second nurses. The horizontal reference line indicates an eigenvalue threshold of 1.

**Figure 5:**
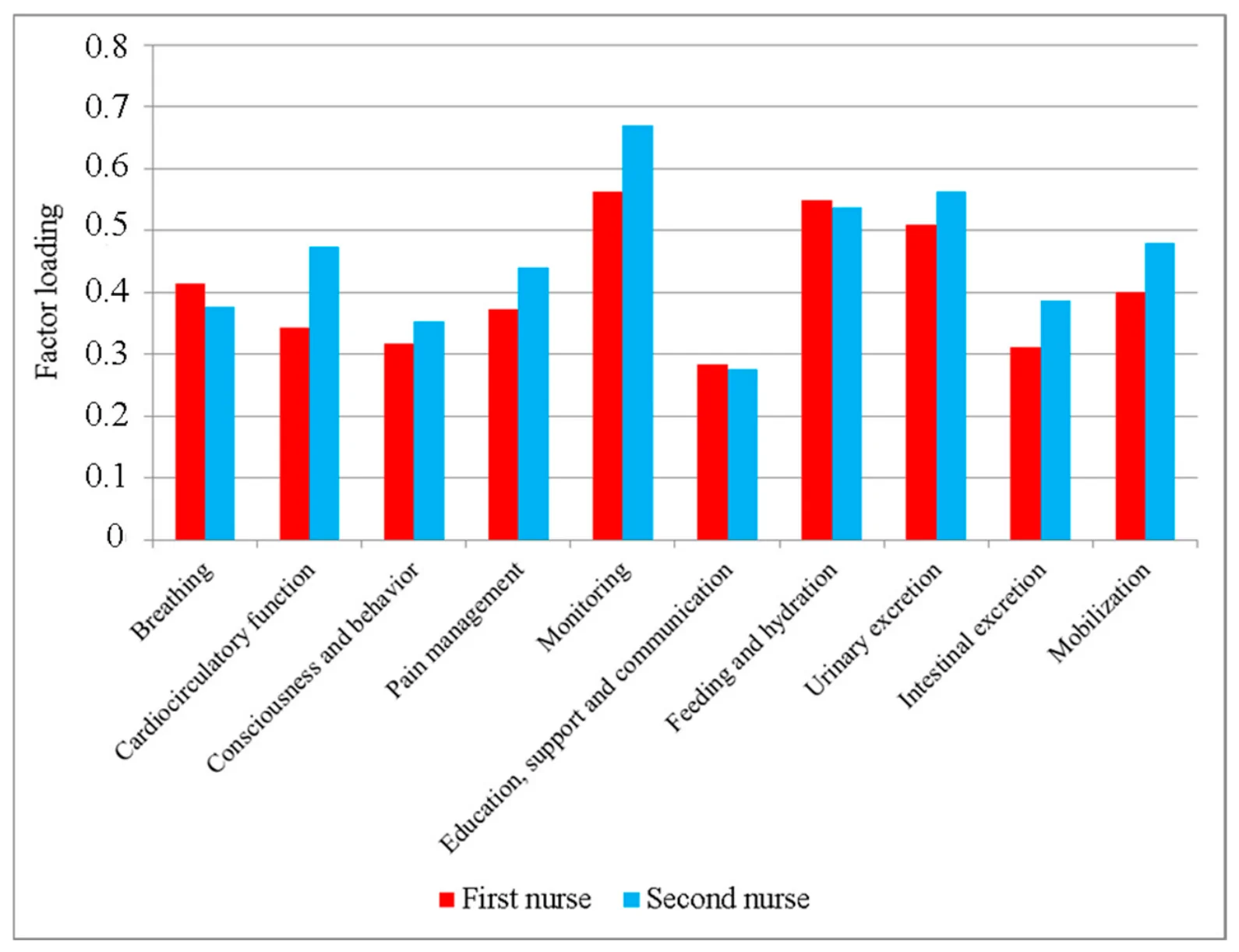
Factor Loadings across the ten GPNS© domains for the first and second nurses.

The KMO measure for data suitability assessment was 0.67 (first nurse) and 0.74 (second nurse). The EFA conducted on assessments from both the first and second nurses in each pair provided evidence consistent with a unidimensional structure of the GPNS©, isolating a single major latent variable conceptualized as “nursing complexity” (Figure 4). The first identified factor accounted for 91.0% and 96.7% of the total variance for the first and second nurses, respectively, further supporting the tool’s unidimensionality. The Monitoring domain demonstrated the highest factor loading (Figure 5).

### 3.6. Completion Time and Perceived Ease of Use

The mean time needed to complete the GPNS© form was 1.8 min (SD = 2.3); the median was 1 min (IQR = 30 s–2 min); the range was 10 s–25 min. Eleven percent of forms required more than 3 min to be completed, and 4.4% exceeded 5 min.

Perceived ease of use was rated as “fairly easy” in 57.6% of assessments and “very easy” in 39.1%, resulting in a high overall positive ease-of-use rating of 96.7%.

## 4. Discussion

Establishing the reliability, validity, and user-friendliness of a measurement instrument is a fundamental prerequisite prior to its implementation in clinical practice [7,14]. To address this requirement, the GPNS© underwent rigorous statistical validation across multiple pediatric care settings after having previously demonstrated face and content validity.

High inter-rater reliability is a necessary prerequisite to support validity, even if it does not single-handedly guarantee it [26]. In line with this, our findings demonstrated strong inter-rater agreement for the overall GPNS© results, qualifying as substantial according to Landis and Koch [27]. At the domain level, the lowest inter-rater agreement was observed in Cardiocirculatory function; Education, support, and communication; and Consciousness and behavior. The variability in Education, support, and communication may stem from the inherent complexity of assessing the therapeutic education needs of both patients and caregivers, alongside unmeasured barriers to compliance (e.g., cultural or linguistic). In the study setting, no standardized tools were in place to objectively quantify the need for therapeutic education, evaluate educational target achievement, or assess the impact of socioeconomic and communication barriers on patient and caregiver engagement. Similarly, validated tools for objectively measuring patient consciousness or behavioral instability were not routinely utilized, likely increasing reliance on individual subjective interpretation. Although inter-rater agreement varied across units, this heterogeneity might be primarily driven by individual nursing perceptions of patient complexity. Crucially, the overall reliability was satisfactory, and the majority of discrepancies occurred between adjacent complexity levels (e.g., white vs. green, green vs. yellow).

The KMO measure for both nurses in each pair indicated an acceptable sampling adequacy (cut-off > 0.50) for the factor analysis [28].

Consistent with a unidimensional factor structure in both the performed analyses, EFA isolated a single underlying latent variable conceptualized as patient “nursing complexity”, thereby supporting the tool’s construct validity [17].

Finally, the results demonstrated a short completion time and very high ease of use for the GPNS© [14]. While completion time exhibited a wide range, a speculative interpretation of this finding is that the extreme maximum value might reflect interruptions during data entry rather than actual completion duration. The median completion time of 1 min, coupled with an overall high positive ease-of-use rating, confirms that the GPNS© is a user-friendly tool for pediatric inpatient settings.

Given the lack of a validated pediatric tool measuring the exact same construct, using nursing clinical judgment as a pragmatic reference standard for concurrent validity was, at the time of the study, the only feasible option, as suggested in the literature [20]. However, agreement between the GPNS© and subjective clinical judgment was rated as fair according to Landis and Koch [27], though it improved when evaluating the weighted Cohen’s kappa index. Several factors may explain this result. First, clinical judgment is inherently subjective and susceptible to cognitive biases [19]. Unpublished exploratory data from a post-validation survey conducted by our research team to explore the root causes of this discrepancy highlighted that nurses frequently incorporated extrinsic organizational factors into their clinical judgment—such as task duration, equipment shortages, perceived workload, or understaffing. Conversely, the GPNS© is strictly patient-centered, focusing on individual nursing needs rather than operational constraints; as Van Slyck [5] emphasized, a patient classification system “is not about the staff; it is about the patients”. Second, given that discrepancies were predominantly observed between adjacent complexity levels, this trend may simply represent borderline cases near the thresholds between adjacent levels rather than substantial misclassification. This interpretation is supported by the higher agreement observed with the weighted Cohen’s kappa index. Furthermore, specific features of the GPNS© may explain why, when compared to subjective clinical judgment, it tended to classify patients into higher nursing complexity levels. Primarily, the tool systematically captures patient nursing needs that intuitive clinical judgment might implicitly take for granted [19]. Moreover, while clinical judgment may encompass a broader assessment of the clinical situation [19], the GPNS© strictly focuses on nursing needs, leading to a more structured and potentially higher complexity level. Thus, rather than indicating an inherent flaw in the tool, this divergence may reflect the fact that intuitive clinical judgment incorporates contextual factors that are intentionally excluded from the GPNS©, which is a patient-centered tool. Therefore, until a suitable instrument becomes available to serve as a validated external criterion for concurrent validation, concurrent validity cannot be definitively established.

Once the concurrent validity of the GPNS© is fully established, its implementation in clinical practice will need to be carefully designed, taking into account its purpose, instructions for use, and the impact of its results (patients’ complexity levels) on decision-making processes, as well as evaluating patient, family, staff, and organizational outcomes.

Ensuring measurement accuracy and minimizing subjective distortion during routine application will require ongoing engagement and periodic refresher training, particularly for newly hired nursing staff [14]. Additionally, to ensure long-term consistency, regular clinical audits should be implemented [8], and longitudinal monitoring should be established to evaluate whether the tool’s strong inter-rater reliability remains consistent over time.

Furthermore, integrating validated objective assessment instruments for specific domains (e.g., therapeutic education requirements or behavioral/consciousness impairment) and establishing standardized nursing care protocols (e.g., defining monitoring frequency based on patient health status) could further enhance the tool’s objectivity.

Although several methodological strategies were implemented to minimize the risk of bias, this study has some limitations that should be considered when interpreting the findings.

First, patients classified within the high-complexity level (red) accounted for less than 1% of total assessments in valid paired forms. This small sample was not sufficient to consider validation as established for this specific subgroup, highlighting the need for further studies involving a larger representative sample of high-complexity patients.

Moreover, the unequal distribution of compiled digital forms across units and the higher prevalence of high complexity level (red) patients among unpaired assessment indicate potential selection bias.

In addition, one of the 17 participating units (Pneumology–Cystic Fibrosis) provided no valid paired assessments, and completion rates varied significantly across the remaining units. The small number of observations in certain units may have contributed to the variability in agreement estimates. The heterogeneity in data collection across units could be largely attributed to differences in unit bed capacity, temporary closures for organizational reasons during the study period, and the exclusion of inpatients aged 18 years and older in units with a higher proportion of adult inpatients, which may have impacted the overall internal validity of the results. Nevertheless, the tool is based on core nursing care needs, which are universal regardless of the specific health condition or unit setting. Notably, nurses from all units of the hospital—including high-intensity care units (where formal validation is currently pending)—actively participated in the tool’s development phase, ensuring that the 10 core nursing needs and their corresponding items reflect the reality of care across the pediatric spectrum.

The fair agreement between GPNS© results and subjective clinical judgment warrants careful consideration. While several potential explanations have been provided, alternative accounts cannot be ruled out, and further concurrent validation using an independent, externally validated criterion—when available—remains necessary to establish concurrent validity.

The same-rater bias due to repeated assessments by the same nurses could not be entirely excluded, although bootstrap analyses indicated a negligible impact on estimated agreement.

Furthermore, potential clustering effects arising from repeated assessments on the same patients were mitigated through clustered bootstrap procedures.

Finally, as this was a single center study, conducted exclusively in low- and medium-intensity care units, the external validity of the GPNS© was not formally established. To evaluate the generalizability and applicability of the tool beyond its development setting [7,13], a multicenter study replicating this methodology across diverse pediatric healthcare environments is currently underway.

Moreover, following the implementation of the GPNS© in clinical practice, future research should investigate the associations between its use and both clinical and organizational outcomes, including clinical risk and quality of care as perceived by nurses, patients, and their caregivers [8,29].

## 5. Conclusions

After having previously established its face and content validity, the GPNS© demonstrated promising psychometric properties. The substantial inter-rater reliability and the results from EFA, consistent with a unidimensional factor structure, along with the short completion time and the very high perceived ease of use, suggest that the GPNS© could be a suitable tool for assessing nursing complexity among hospitalized pediatric patients in low- and medium-intensity care units within its development setting. However, because concurrent validity cannot yet be considered established, further validation studies against an independent, externally validated criterion remain necessary. Furthermore, the paucity of patients classified within the high-complexity level was not sufficient to consider validation for this subgroup as fully established, requiring further research to assess a larger, representative sample of these patients.

## 6. Copyright

The Gaslini Pediatric Nursing Score© is protected by copyright (SIAE—Italian Society of Authors and Publishers, Reg. No. 2022/00873, Year 2022). The copyright is held by IRCCS Istituto Giannina Gaslini, to which the author Silvia Scelsi assigned all intellectual property rights. Although the complete GPNS© is published [15], permission for free use or adaptation, along with its instructions for use and the images of the digital interface, must be requested from the Technology Transfer Office of IRCCS Istituto Giannina Gaslini (e-mail: tto@gaslini.org).

## Figures and Tables

**Table 1:** Agreement on GPNS© complexity levels between nurse pairs.

	Second Nurse				
First Nurse	White	Green	Yellow	Red	Total
White	104	6	3	0	113
Green	14	155	11	2	182
Yellow	1	19	90	1	111
Red	0	0	0	1	1
Total	119	180	104	4	407

Observed agreement: 86.0%; Expected agreement: 34.9%; Unweighted Cohen’s kappa = 0.785 (95% CI: 0.700–0.870); Weighted Cohen’s kappa = 0.839 (95% CI: 0.775–0.904).

**Table 2:** Inter-rater agreement for overall clinical judgment between nurse pairs.

	Second Nurse				
First Nurse	White	Green	Yellow	Red	Total
White	195	13	3	1	212
Green	15	99	15	3	132
Yellow	1	19	41	1	62
Red	0	1	0	0	1
Total	211	132	59	5	407

Observed agreement: 82.3%; Expected agreement: 39.7%; Unweighted Cohen’s kappa= 0.706 (95% CI: 0.601–0.812); Weighted Cohen’s kappa = 0.776 (95% CI: 0.676–0.877).

**Table 3:** Agreement between GPNS© results and overall clinical judgment.

	GPNS©				
Clinical Judgment	White	Green	Yellow	Red	Total
White	210	185	27	1	423
Green	16	142	106	0	264
Yellow	6	34	79	2	121
Red	0	1	3	2	6
Total	232	362	215	5	814

Observed agreement: 53.2%; Expected agreement: 33.2%; Unweighted Cohen’s kappa = 0.300 (95% CI: 0.223–0.376); Weighted Cohen’s kappa = 0.523 (95% CI: 0.454–0.593).

## Data Availability

The data supporting the findings of this study are available upon reasonable request from the corresponding author.

## Supplementary Materials

The following supporting information can be downloaded at: https://www.mdpi.com/article/10.3390/children13101370/s1, Table S1: STROBE Statement—checklist of items that should be included in reports of observational studies.

## Abbreviations

The following abbreviations are used in this manuscript:

CI	Confidence Interval
EFA	Exploratory Factor Analysis
GPNS©	Gaslini Pediatric Nursing Score©
I-CVI	Item-Content Validity Index
IQR	Interquartile Range
IRB	Institutional Review Board
NPCS	Nursing Patient Classification System
S-CVI/Ave	Scale-Content Validity Index, Average
S-CVI/UA	Scale-Content Validity Index, Universal Agreement
SD	Standard Deviation
